# Controlling Effects of Irradiance and Heterotrophy on Carbon Translocation in the Temperate Coral *Cladocora caespitosa*

**DOI:** 10.1371/journal.pone.0044672

**Published:** 2012-09-10

**Authors:** Pascale Tremblay, Christine Ferrier-Pagès, Jean François Maguer, Cécile Rottier, Louis Legendre, Renaud Grover

**Affiliations:** 1 Ecophysiology Team, Centre Scientifique de Monaco, Monaco, Principality of Monaco; 2 LEA CSM-CNRS ‘BIOSENSIB’, Monaco, Principality of Monaco; 3 LEMAR - UMR 6539 UBO/CNRS/IRD, Institut Universitaire Européen de la Mer, Plouzané, France; 4 Université Pierre et Marie Curie, UMR 7093, Laboratoire d’Océanographie de Villefranche, Villefranche-sur-Mer, France; 5 CNRS, UMR 7093, Laboratoire d’Océanographie de Villefranche, Villefranche-sur-Mer, France; Institute of Marine Research, Norway

## Abstract

Temperate symbiotic corals, such as the Mediterranean species *Cladocora caespitosa*, live in seasonally changing environments, where irradiance can be ten times higher in summer than winter. These corals shift from autotrophy in summer to heterotrophy in winter in response to light limitation of the symbiont’s photosynthesis. In this study, we determined the autotrophic carbon budget under different conditions of irradiance (20 and 120 µmol photons m^−2^ s^−1^) and feeding (fed three times a week with *Artemia salina* nauplii, and unfed). Corals were incubated in H^13^CO_3_^−^-enriched seawater, and the fate of ^13^C was followed in the symbionts and the host tissue. The total amount of carbon fixed by photosynthesis and translocated was significantly higher at high than low irradiance (ca. 13 *versus* 2.5–4.5 µg cm^−2^ h^−1^), because the rates of photosynthesis and carbon fixation were also higher. However, the percent of carbon translocation was similar under the two irradiances, and reached more than 70% of the total fixed carbon. Host feeding induced a decrease in the percentage of carbon translocated under low irradiance (from 70 to 53%), and also a decrease in the rates of carbon translocation per symbiont cell under both irradiances. The fate of autotrophic and heterotrophic carbon differed according to irradiance. At low irradiance, autotrophic carbon was mostly respired by the host and the symbionts, and heterotrophic feeding led to an increase in host biomass. Under high irradiance, autotrophic carbon was both respired and released as particulate and dissolved organic carbon, and heterotrophic feeding led to an increase in host biomass and symbiont concentration. Overall, the maintenance of high symbiont concentration and high percentage of carbon translocation under low irradiance allow this coral species to optimize its autotrophic carbon acquisition, when irradiance conditions are not favourable to photosynthesis.

## Introduction

Many scleractinian corals live in symbiosis with photosynthetic dinoflagellates, commonly called zooxanthellae, that translocate most of the photosynthesized compounds to the coral host [1], [2]. These carbon-based compounds consist mainly of polysaccharides, lipids and amino acids [3]–[7], which are either rapidly respired by the coral host for its basic metabolic needs, or stored as long-term reserves [8]–[10]. Tropical coral species, which thrive all year round in well-lit environments, have high rates of photosynthesis, and high production and translocation of photosynthates to the host. The photosynthates satisfy most of the holobiont daily requirements [1], [9], [11], the remaining needs being satisfied by heterotrophic feeding or catabolism of stored lipid [2], [12], [13]. In contrast, in temperate species such as the Mediterranean symbiotic coral *Cladocora caespitosa* (Linnaeus, 1767), symbiont productivity is limited during winter by low underwater irradiance and seawater temperature [14], [15]. In the Ligurian Sea (Northwestern Mediterranean) at 7–15 m depth where these corals are abundant, temperature increases from 12°C in winter to ca. 22 to 24°C in summer, and daily mean irradiance increases from less than 20 µmol photons m^−2^ s^−1^ to ten times that value [16]–[18]. Food availability at that same depth also varies with seasons, i.e. concentrations of particulate and dissolved organic matter are high in winter, and lower in summer because density stratification of the water column prevents vertical mixing [19], [20]. These important environmental changes force the coral host and its symbionts to continuously acclimatize, i.e. to optimize their acquisition of inorganic or organic nutrients *via* autotrophy or heterotrophy.

The biology of *C. caespitosa* has been well studied [21] because it is one of the main endemic symbiotic coral in the Mediterranean Sea, with a wide distribution in both the eastern and western basins [22]–[25]. That species is a good model for studying the functioning of temperate symbiotic organisms [26], and it also represents a potential climate archive for the Mediterranean region. The responses of the holobiont to changes in irradiance and food concentration have been studied in the laboratory and *in situ*, showing that *C. caespitosa* had high rates of grazing of living particles compared to tropical scleractinian corals [27], especially under low irradiance [28], and mostly relied on heterotrophy during winter [29]. As for many other scleractinian coral species (see review by [30]), feeding increased both the skeletal growth rate and the symbiont concentration, particularly at low temperature [31]. Also, variations in irradiance induced only small changes in symbiont concentration [31], [32], which remained high even when corals were maintained in darkness during several weeks [28]. This feature has also been observed in some other temperate cnidarian symbioses, i.e. algae-sea anemones (reviewed in [14]).

The maintenance of constantly high symbiont concentrations within the tissue of *C. caespitosa,* independently of the irradiance, is puzzling. This feature could be either a strategy to maximise the photosynthetic rate and acquisition of carbon under low irradiance [15], [33], or a lack of host regulation of the symbionts, which would function in a parasitic way [34], [35]. In the first case, symbionts would have the same function under low and high light, i.e. they would maintain high rates of photosynthesis and carbon translocation to their host, whereas in the second case, they would retain the photosynthesized carbon for their own needs or acquire carbon through the host heterotrophy.

Differently from symbionts associated to tropical species, which most often have translocation rates as high as 90% of the fixed carbon [1], [2], [12], translocation from temperate symbionts seems to vary widely depending on irradiance, or on the symbiont or host species. Translocation rates estimated in the few studies performed on temperate sea anemones and zoanthids range from 32% of the fixed carbon in *Anemonia viridis* to 96% in *Isozoanthus sulcatus*, the two species being maintained under the same low irradiance of 10 µmol photons m^−2^ s^−1^ [36], and from 30 to 90% for the sea anemone *Anthopleura elegantissima* [37], [38]. More investigations are thus needed to elucidate the role of temperate symbionts in the carbon budget of their animal cnidarian hosts.

Given the present and upcoming changing conditions of irradiance and food supply in many temperate marine environments, key questions concerning the functioning of temperate coral symbioses are: (1) What are the carbon translocation rates of temperate symbionts, i.e. what is the contribution of symbionts to the nutritional needs of their coral hosts? (2) How much of the photosynthetically-acquired carbon is retained within the symbiotic association, and how much is lost as respiratory CO_2_ and particulate (POC) or dissolved (DOC) organic carbon? (3) How do external factors such as irradiance and food supply affect carbon fixation, translocation, and utilization by the symbiotic association? Responses to these questions are needed to understand the role of symbionts in the functioning of their temperate host, and the importance of autotrophic compounds in maintaining coral metabolism under conditions of changing irradiance and heterotrophic inputs.

In the present study, we addressed the above questions using a new model of carbon translocation based on the ^13^C tracer [39], with which we estimated both the amount of photosynthetically-fixed carbon translocated from the symbionts to the host and the fate of this translocated carbon in colonies of the temperate scleractinian coral *C. caespitosa*. We hypothesised that carbon translocation rates would be low (1) under low irradiance because of carbon limitation of the symbionts, and/or (2) when the hosts are fed because of carbon sufficiency of the host.

**Table 1:** Experimental setup of nubbins of the scleractinian coral *C. caespitosa* in eight tanks (numbered from 1 to 8).

	20 µmol photons m^−2^ s^−1^				120 µmol photons m^−2^ s^−1^				
	Fed (LIF)		Unfed (LIU)		Fed (HIF)		Unfed (HIU)		
Parameters	Tank 1	Tank 2	Tank 3	Tank 4	Tank 5	Tank 6	Tank 7	Tank 8	Total
Calcification rate and protein concentration	3	3	3	3	3	3	3	3	24
Photosynthesis and respiration rate, symbiontand chlorophyll concentration	3	3	3	3	3	3	3	3	24
Respiration rate of symbionts, and symbiont concentration	3	3	3	3	3	3	3	3	24
^13^C experiments and C:N ratio	9	9	9	9	9	9	9	9	72
Total per tank	18	18	18	18	18	18	18	18	
Total per condition	36		36		36		36		144

There were three colonies and 48 nubbins per colony (six nubbin per colony, i.e. 18 nubbins, in each tank), for a total of 144 nubbins. Each nubbin was used for one type of measurement.

## Materials and Methods

### Experimental Design

Three large colonies of the Mediterranean scleractinian coral *C. caespitosa* (Faviiidae), collected in the Bay of Fiascherino in the Gulf of La Spezia (Italy, 44°03′N, 9°55′E) at 7–10 m depth, under collection permit DPR 9/6/1076 N. 1057 from the Ministry of Agriculture, Food and Forestry of Italy, were used for this experiment. Colonies were cleaned of epiphytes, and each divided into nubbins of 3–4 polyps. A total of 144 nubbins were prepared and maintained (under the control conditions described below) for four weeks until they recovered. During this period, the nubbins were fed twice a week with *Artemia salina* nauplii. The holding tanks were in an open flow system (renewal rate of 10 l h^−1^), at a temperature kept constant at 18.0±0.5°C using heaters connected to electronic controllers, and under an irradiance of 50 µmol photons m^−2^ s^−1^ (12 h light:12 h dark photoperiod). Seawater contained low levels of inorganic and organic nutrients [40].

**Table 2:** List of symbols, definition and units.

Symbol	Definition
C	Carbon
*C_C_*	C used by calcification (µg C cm^−2^ h^−1^)
*C_inc_*	^13^C enrichment of the incubation medium (%)
*C_L_*	Amount of C lost (µg C cm^−2^ h^−1^ or %)
*C_meas_*	^13^C measured in the sample (%)
*C_nat_*	Natural abundance in ^13^C in control nubbins (%)
*C_R_*	Percentage of fixed C remaining in symbionts, host tissue and POC (%)
*M_C_*	Mass of C per milligram of tissue or symbionts (µg mg^−1^) or released POC (µg)
*M_sample_*	Mass of the freeze-dried sample (mg)
*M_Sk_*	CaCO_3_ produced by calcification (µg CaCO_3_ cm^−2^ h^−1^)
*P_C_*	Gross C fixed photosynthetically by symbionts (µg C cm^−2^ h^−1^)
*P_g_*	Oxygen produced by gross photosynthesis (µmol O_2_ cm^−2^ h^−1^)
*P_n_*	Oxygen produced by net photosynthesis (µmol O_2_ cm^−2^ h^−1^)
*PQ*	Photosynthetic quotient (equal to 1.1 mol O_2_:mol C)
*R*	Oxygen consummed by respiration of holobiont (µmol O_2_ cm^−2^ h^−1^)
*R_C_*	C respired by holobiont (µg C cm^−2^ h^−1^)
*R_H_*	C respired by coral host (µg C cm^−2^ h^−1^)
*RQ*	Respiratory quotient (equal to 0.8 mol O_2_:mol C)
*R_S_*	C respired by symbionts (µg C cm^−2^ h^−1^)
*S*	Nubbin surface area (cm^2^)
*T_S_*	Amount of C translocated calculated from the symbiont rates (µg C cm^−2^ h^−1^ or %)
*t_chase_*	Incubation time of the nubbins in the non-enriched incubation medium in the light (h)
*t_pulse_*	Incubation time of the nubbins in the enriched incubation medium (h)
*ρ_DOC_*	C incorporation rate in released DOC (not measured)
*ρ_H_*	C incorporation rate in coral tissue (µg C cm^−2^ h^−1^)
*ρ_POC_*	C incorporation rate in released POC (µg C cm^−2^ h^−1^)
*ρ_S_*	C incorporation rate in symbiont (µg C cm^−2^ h^−1^)

After healing, the 48 nubbins from each colony were equally divided among eight 20 l tanks (six nubbins per colony in each tank, i.e. 18 nubbins per tank; Table 1) corresponding to four treatments (duplicated tanks): Low Irradiance of 20 µmol photons m^−2^ s^−1^, Fed (LIF) and Unfed (LIU); and High Irradiance of 120 µmol photons m^−2^ s^−1^, Fed (HIF) and Unfed (HIU). The irradiance treatment was a 12 h light:12 h dark photoperiod, and fed nubbins were given *A. salina* nauplii three times a week. Corals were maintained five weeks under the above experimental conditions, after which several parameters were measured as explained below.

### Rates of Calcification, Photosynthesis and Respiration

Calcification rates of six nubbins per treatment (one from each colony in each tank, i.e. 24 nubbins; Table 1) were measured at the culture’s irradiance using the alkalinity anomaly technique [41]. To do so, nubbins were incubated for 6 h in close beakers filled with 200 ml of 0.45 µm-filtered seawater (FSW), continuously stirred with a stirring bar and maintained at 18.0±0.5°C. At the end of the incubation, nubbins were frozen for later determination of protein content after extraction in 1 M NaOH at 90°C during 30 min, using the BCAssay Kit (Interchim, Montluçon, France) [42] and a spectrofluorometer Xenius® (Safas, Monaco). For total alkalinity (TA), 60 ml seawater were sampled at the beginning and the end of incubation, filtered on a 0.2 µm syringe filter (Minisart, #16532, Sartorius Stedim Biotech, Germany), preserved with 20 µl of 50% mercuric chloride (HgCl_2_) to avoid biological alteration, and stored at 4°C pending rapid analysis. TA was determined on triplicated 16-ml subsamples using a Metrohm 888 Titrando system, with a pH electrode calibrated on the National Institute of Standards and Technology scale. Changes in TA during incubation were used to estimate the calcification rates [41] expressed as µmol CaCO_3_ cm^−2^ h^−1^. The amount of carbon used for calcification was calculated according to: *C_C_* = *M_Sk_*×12/100, where *M_Sk_* is the µg CaCO_3_ produced and 12/100 is the ratio of molecular masses of C (12) and CaCO_3_ (100). All data were normalized to the skeletal surface area, determined according to Rodolfo-Metalpa et al. [43].

**Figure 1:**
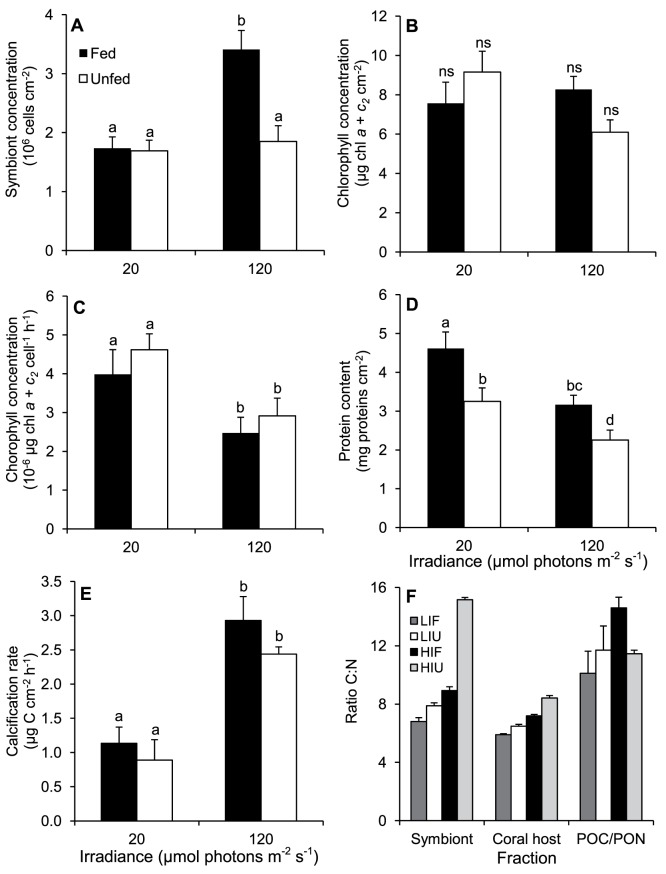
Effect of irradiance and heterotrophy on the main physiological parameters of *C. caespitosa*. (A) Symbiont concentration, (B) chlorophyll concentration per skeletal surface, (C) chlorophyll concentration in symbiont cells, (D) protein content, (E) calcification rate, and (F) C:N ratios for symbionts, coral host, and released POC/PON, for fed and unfed nubbins maintained at low and high irradiances. Data are means ± standard errors of means of *n* = 12 (A), *n* = 6 (B to E), *n* = 23 (F, symbionts and host), or *n* = 3 (F, POC/PON) measurements. Bars with different letters (a to d) are significantly different.

**Figure 2:**
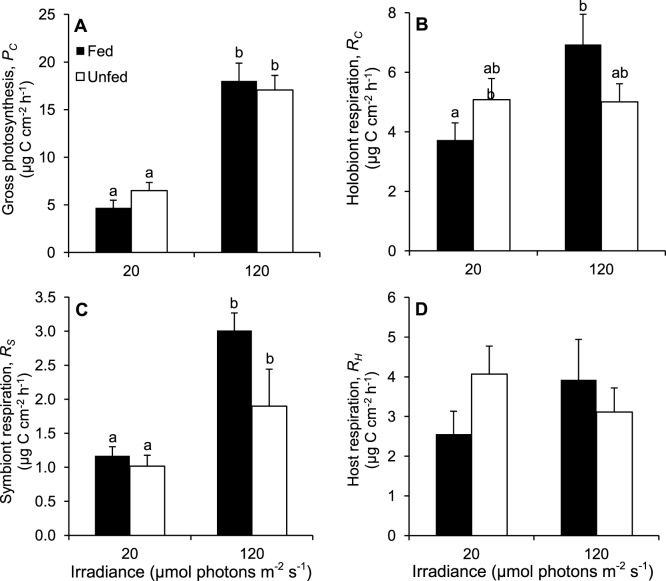
Effect of irradiance and heterotrophy on the rates of photosynthesis and respiration of *C. caespitosa*. (A) Gross photosynthesis, *P_C_*, (B) holobiont respiration, *R_C_*, (C) symbiont respiration, *R_S_*, and (D) host respiration, *R_H_*, for fed and unfed nubbins maintained at low and high irradiances. Data are means ± standard errors of means of *n* = 6 measurements. Bars with different letters (a and b) are significantly different.

**Table 3:** Results of the statistical analyses on the effects of irradiance and heterotrophy on the main physiological parameters of *C. caespitosa.*

Factor	Degrees of freedom	*p*	*F* value
*Symbiont concentration*			
Irradiance	1	**0.0005**	14.38
Feeding mode	1	**0.0029**	9.94
Irradiance*Feeding mode	1	**0.0047**	8.85
Error	44	–	–
*Chlorophyll concentration per surface area*			
Irradiance	1	0.1790	1.94
Feeding mode	1	0.5950	0.29
Irradiance*Feeding mode	1	**0.0227**	6.09
Error	20	–	–
*Chlorophyll concentration per symbiont cell*			
Irradiance	1	**0.0034**	11.00
Feeding mode	1	0.2818	1.22
Irradiance*Feeding mode	1	0.8424	0.04
Error	20	–	–
*Protein concentration*			
Irradiance	1	**0.0012**	14.31
Feeding mode	1	**0.0015**	13.52
Irradiance*Feeding mode	1	0.9988	<0.01
Error	20	–	–
*Calcification rate*			
Irradiance	1	**<0.0001**	39.98
Feeding mode	1	0.1802	1.93
Irradiance*Feeding mode	1	0.6458	0.23
Error	20	–	–

Results of factorial analyses of variance (ANOVA) for symbiont (*n* = 12), chlorophyll (*n* = 6) and protein (*n* = 6) concentrations, and calcification rate (*n* = 6), with two factors (irradiance, and feeding mode). Chlorophyll concentration per surface area and protein concentration were ln-transformed prior to analysis. Significant *p*-values are in bold.

**Table 4:** Results of the statistical analyses on the effects of irradiance and heterotrophy on the C:N ratio and the natural percentage of ^13^C of *C. caespitosa*.

Factor	Degrees of freedom	*p*	*F* or *H* value
*C:N ratio of symbionts (Sheirer-Ray-Hare)*			
Irradiance	1	**<0.0001**	62.85
Feeding mode	1	**<0.0001**	25.55
Irradiance*Feeding mode	1	0.1389	2.19
Error	88	–	–
*C:N ratio of coral host (ANOVA)*			
Irradiance	1	**<0.0001**	145.06
Feeding mode	1	**<0.0001**	41.88
Irradiance*Feeding mode	1	0.1277	2.36
Error	88	–	–
*C:N ratio of released POC/PON (Sheirer-Ray-Hare)*			
Irradiance	1	0.0561	3.65
Feeding mode	1	0.8065	0.06
Irradiance*Feeding mode	1	**0.0318**	4.61
Error	7	–	–
*Natural percentage of ^13^C in symbionts (ANOVA)*			
Irradiance	1	0.8626	0.03
Feeding mode	1	0.9173	0.01
Irradiance*Feeding mode	1	0.1930	1.90
Error	12	–	–
*Natural percentage of ^13^C in coral host (ANOVA)*			
Irradiance	1	0.2809	1.27
Feeding mode	1	0.4295	0.67
Irradiance*Feeding mode	1	0.1980	1.86
Error	12	–	–

Results of Scheirer-Ray-Hare’s tests (*H* values) for the C:N ratio of symbionts (*n* = 23) and released POC/PON (*n* = 3), and of factorial analysis of variance (ANOVA, *F* values) for the C:N ratio of coral host (*n* = 23) and for natural percentage of ^13^C (measured in non-enriched control samples, *n* = 3), with two factors (irradiance, and feeding mode). C:N values of coral host were ln-transformed prior to analysis. Significant *p*-values are in bold.

**Table 5:** Results of the statistical analyses on the effects of irradiance and heterotrophy on the photosynthesis and respiration rates of *C. caespitosa*.

Factor	Degrees of freedom	*p*	*F* value
*Gross photosynthesis (P_C_)*			
Irradiance	1	**0.0000**	79.53
Feeding mode	1	0.7357	0.10
Irradiance*Feeding mode	1	0.3174	1.05
Error	20	–	–
*Holobiont respiration (R_C_)*			
Irradiance	1	**0.0487**	4.41
Feeding mode	1	0.7078	0.14
Irradiance*Feeding mode	1	**0.0401**	4.82
Error	20	–	–
*Symbiont respiration (R_S_)*			
Irradiance	1	**0.0004**	18.24
Feeding mode	1	0.0612	3.93
Irradiance*Feeding mode	1	0.1481	2.26
Error	20	–	–
*Host respiration (R_H_)*			
Irradiance	1	0.7834	0.08
Feeding mode	1	0.6464	0.22
Irradiance*Feeding mode	1	0.1355	2.42
Error	20	–	–

Results of factorial analyses of variance (ANOVA) for gross photosynthesis (*P_C_*), holobiont respiration (*R_C_*), symbionts respiration (*R_S_*), and host respiration (*R_H_*), with two factors (irradiance, and feeding mode); *n* = 6 replicates. Significant *p*-values are in bold.

Rates of respiration (*R*) and net photosynthesis (*P_n_*) were assessed at 0, 20, and 120 µmol photons m^−2^ s^−1^ on six nubbins per treatment (one from each colony in each tank, i.e. 24 nubbins, Table 1) using the respirometry technique [33]. Rates of gross photosynthesis (*P_g_*) were calculated by adding *R* to *P_n_*. Samples were frozen for the later determination of symbiont and chlorophyll (chl) concentrations, according to Rodolfo-Metalpa et al. [43] for symbionts and Jeffrey and Humphrey [44] for chl *a* and *c_2_*. Data were normalised to the skeletal surface area of each nubbin (µmol O_2_ cm^−2^ h^−1^).

In addition to the above measurements, the respiration rates of freshly isolated symbionts were also determined on six nubbins per treatment (one per colony in each tank, i.e. 24 nubbins; Table 1). Symbionts were extracted in FSW using an air-brush, homogenised, and centrifuged at 850 g for 10 min. The pellet containing the symbionts was resuspended in FSW. Respiration rates and symbiont concentration were measured as described above.

**Figure 3:**
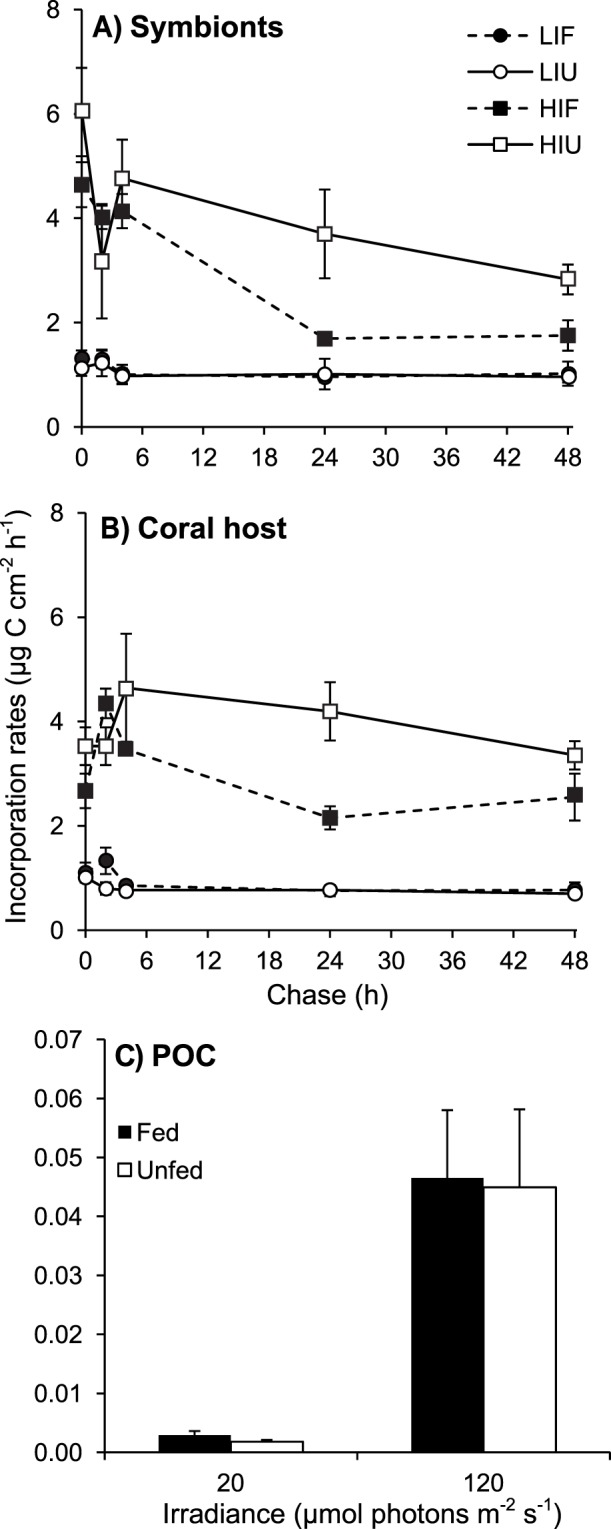
Effect of irradiance and heterotrophy on the carbon incorporation rates of *C. caespitosa*. Carbon incorporation rates (*ρ*) in (A) symbionts and (B) coral host over the 48-h chase, and (C) in released POC at 24 h of chase, for fed and unfed nubbins maintained at low and high irradiances. Data represent means and standard errors of means of *n* = 3 measurements.

**Table 6:** Results of the statistical analyses on the effects of irradiance and heterotrophy on the model estimates of carbon translocation of *C. caespitosa*.

Factor	Degrees offreedom	At the beginning (0 h)			At the end (48 h)	
		*p*		*F* value	*p*	*F* value
*Incorporation rate in symbionts (ρ_S_)*						
Irradiance	1	**<0.0001**		71.06	**0.0006**	29.35
Feeding mode	1	0.2469		1.56	0.0677	4.46
Irradiance*Feeding mode	1	0.1474		2.57	**0.0468**	5.51
Error	8	–		–	–	–
*Incorporation rate in coral host (ρ_H_)*						
Irradiance	1	**0.0001**		58.94	**<0.0001**	65.84
Feeding mode	1	0.1851		2.10	0.2202	1.77
Irradiance*Feeding mode	1	0.1114		3.20	0.1486	2.55
Error	8	–		–	–	–
*Fixed carbon remaining in symbionts (C_R_)*						
Irradiance	1	0.0531		5.14	0.1058	3.32
Feeding mode	1	0.9184		0.01	0.9818	<0.01
Irradiance*Feeding mode	1	**0.0207**		8.25	**0.0409**	5.93
Error	8	–		–	–	–
*Fixed carbon remaining in coral host (C_R_)*						
Irradiance	1	0.4868		0.53	0.1666	2.32
Feeding mode	1	0.6899		0.17	0.9643	<0.01
Irradiance*Feeding mode	1	**0.0266**		7.35	**0.0341**	6.51
Error	8	–		–	–	–
*Amount of lost carbon (C_L_ in* µ*g C)*						
Irradiance	1	**<0.0001**		87.41	**<0.0001**	318.91
Feeding mode	1	0.3843		0.85	0.3717	0.90
Irradiance*Feeding mode	1	**0.0028**		18.03	**0.0010**	25.28
Error	8	–		–	–	–
*Percent of lost carbon (C_L_ in %)*						
Irradiance	1	0.2714		1.40	0.7108	0.15
Feeding mode	1	0.7885		0.08	0.9765	0.00
Irradiance*Feeding mode	1	**0.0108**		10.90	**0.0313**	6.79
Error	8	–		–	–	–
*Amount of photosynthate translocation (T_S_ in µg C)*						
Irradiance	1		**<0.0001**	167.64	**<0.0001**	1441.50
Feeding mode	1		0.4109	0.75	0.0611	4.74
Irradiance*Feeding mode	1		**0.0103**	11.14	**0.0004**	34.24
Error	8		–	–	–	–
*Percent of photosynthate translocation (T_S_ in %)*						
Irradiance	1		0.6766	0.19	**0.0037**	16.41
Feeding mode	1		0.0546	5.06	**0.0296**	6.98
Irradiance*Feeding mode	1		**0.0093**	11.59	**0.0149**	9.54
Error	8		–	–	–	–

Results of factorial analyses of variance (ANOVA) for model estimates of carbon translocation at the beginning (0 h) and the end (48 h) of the chase interval, with two factors (irradiance, and feeding mode); *n* = 3 replicates. Significant *p*-values are in bold.

The autotrophic carbon acquired (*P_C_*) and respired (*R_C_*) were calculated for each treatment by converting the oxygen values to carbon equivalents using the molar weights: *P_C_* = *P_g_*×12/*PQ* and *R_C_* = *R*×12×*RQ* [45], where *PQ* and *RQ* are photosynthetic and respiratory quotients equal to 1.1 mol O_2_:mol C and 0.8 mol C:mol O_2_, respectively [1].

**Figure 4:**
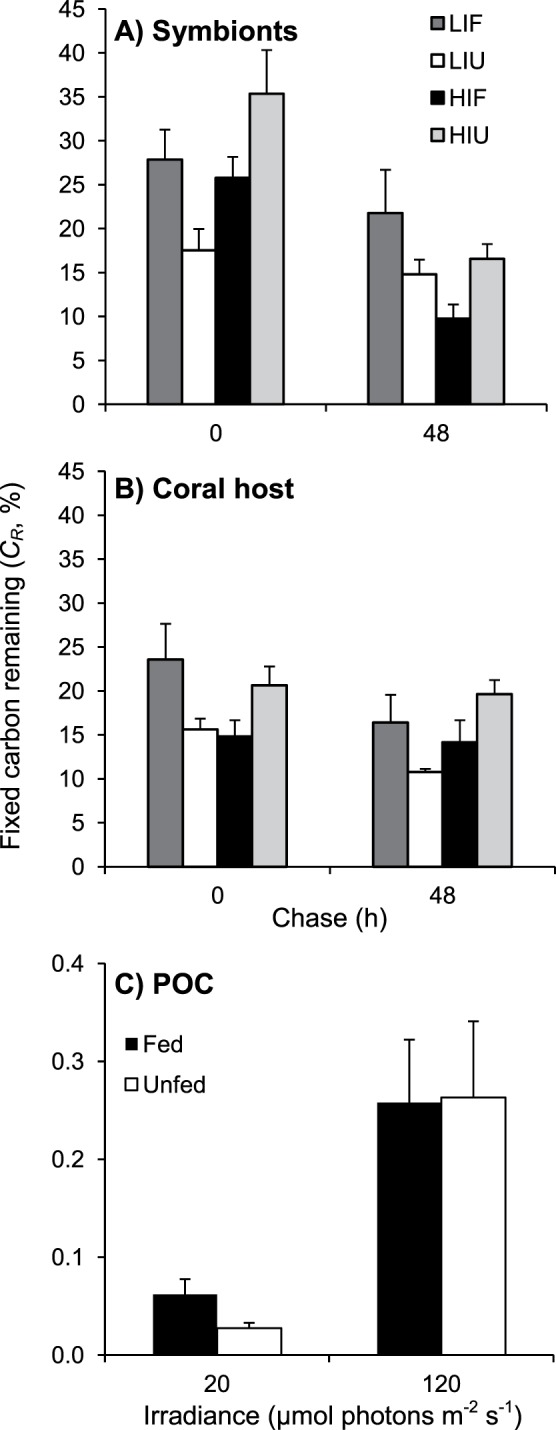
Effect of irradiance and heterotrophy on the percentage of fixed carbon remaining in *C. caespitosa*. Percentage of fixed carbon that remained (*C_R_*) in (A) symbionts and (B) host after 0 and 48 h, and (C) in released POC after 24 h, for fed and unfed nubbins maintained at low and high irradiances. Data represent means and standard errors of means of *n* = 3 measurements.

**Figure 5:**
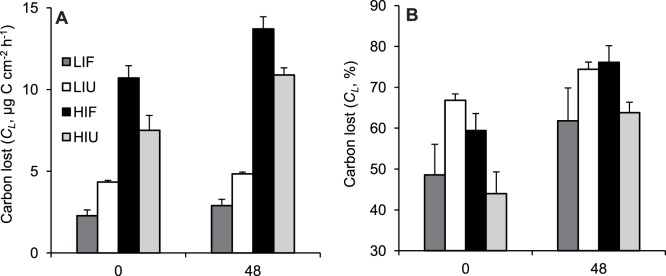
Effect of irradiance and heterotrophy on the carbon lost in *C. caespitosa*. (A) Amount and (B) percentage of carbon lost by the holobiont (*C_L_*), for fed and unfed nubbins maintained at low and high irradiances. Data represent means and standard errors of means of *n* = 3 measurements.

### H^13^CO_3_ Labelling Experiments

H^13^CO_3_ labelling experiments were performed on the nine remaining nubbins per tank (i.e. 18 nubbins per experimental condition for a total of 72 nubbins; Table 1), according to Tremblay et al. [39]. Briefly, corals were placed in H^13^CO_3_^−^ (NaH^13^CO_3_ 98 atom %^13^C, #372382, Sigma-Aldrich, St-Louis, MO, USA) enriched seawater, and %^13^C was measured at the end of the incubation in the symbionts, coral tissue, and released mucus (i.e. POC/PON). For each experimental condition, 15 beakers were filled with 200 ml FSW, enriched with a concentration of 0.6 mM NaH^13^CO_3_ (corresponding to a 23% ^13^C enrichment of the incubation medium). Fifteen nubbins (five nubbins per colony) were incubated individually during 5 h in the ^13^C-enriched FSW maintained at 18.0±0.5°C, after which they were transferred in 15 other beakers containing non-enriched FSW (chase). Three nubbins (one per colony) were removed after 0, 2, 4, 24, and 48 h, and frozen immediately at −20°C. To have an estimate of the mean daily POC production by these corals, seawater samples were taken after 24 h. This seawater, containing the released POC/PON, was filtered onto 25 mm pre-combusted GF/F glass microfiber filters (#1825-025, Whatman). The filters were treated with 10% HCl, rinsed with distilled water, and dried at 60°C, as in Tremblay et al. [27]. Three control nubbins per condition (one per colony, incubated from the beginning in 200 ml non-enriched seawater) were ran in parallel and sampled after 0, 24, and 48 h. At the end of incubation, the nubbins were frozen immediately at −20°C until analysis. The above incubations were repeated for the four experimental conditions.

**Figure 6:**
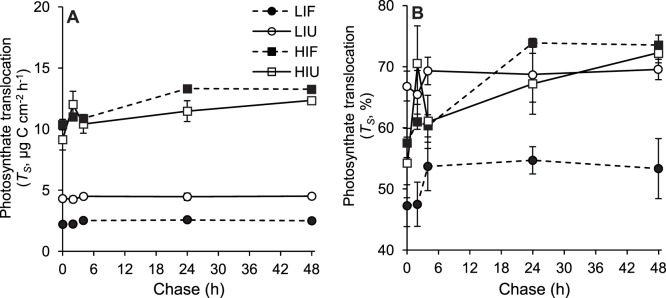
Effect of irradiance and heterotrophy on the carbon translocated in *C. caespitosa*. (A) Amount per skeletal surface and (B) percentage of photosynthesized carbon translocated to the host by symbionts (*T_S_*), for fed and unfed nubbins maintained at low and high irradiances. Data represent means and standard errors of means of *n* = 3 measurements.

All nubbins were treated according to Tremblay et al. [39]. Briefly, tissue was detached from the skeleton in FSW using an air-brush. The slurry was homogenised, and the animal and symbiont fractions separated by centrifugation. Samples were flash-frozen in liquid nitrogen, and freeze-dried until analysis. The %^13^C, and the carbon and nitrogen content of the animal tissue, symbionts, and released POC/PON were determined with a mass spectrometer (Delta Plus, Thermofisher Scientific, Bremen, Germany) coupled with a C/N analyzer (Flash EA, Thermofisher Scientific).

**Figure 7:**
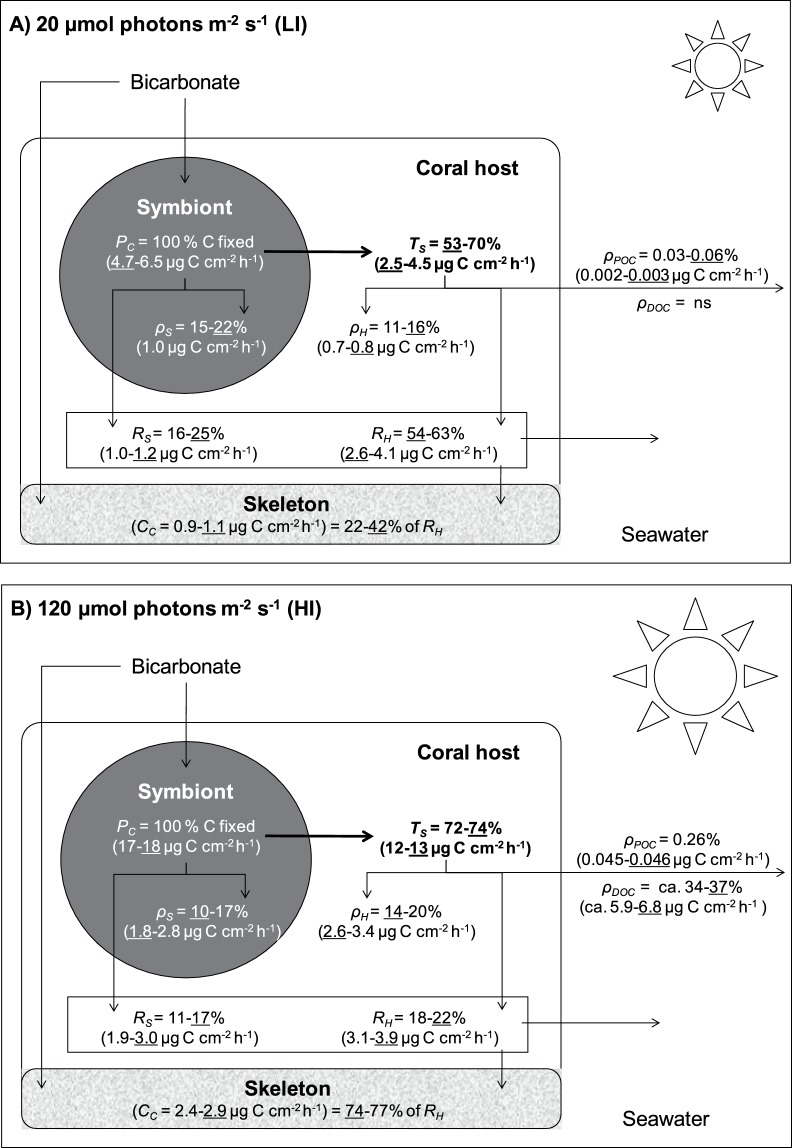
Mass-balanced results of photosynthate translocation and carbon budget in *C. caespitosa*. Results under (A) low (LI) and (B) high irradiances (HI), based on ^13^C experiments after 48 h of incubation (or 24 h for POC). Underlined values are for fed colonies. Symbols are defined in the text and summarised in Table 2. Data represent means and standard errors of means of *n* = 3 measurements for ^13^C-based rates (*ρ_S_*, *ρ_H_*, *ρ_POC_*, *ρ_DOC_*, and *T_S_*) and *n* = 6 measurements for rates of photosynthesis (*P_C_*), respiration (*R_S_*, and *R_H_*) and calcification (*C_C_*).

### Autotrophic Carbon Budget Calculations

The equations used to calculate autotrophic carbon budget are fully described in Tremblay et al. [39], and are only listed here. The carbon incorporation rate (*ρ*) in the symbionts (*ρ_S_*), animal tissue (*ρ_H_*) and released POC (*ρ_POC_*), expressed in µg C cm^−2^ h^−1^, was calculated as follow:
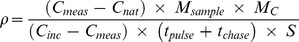
(1)where *C_meas_* and *C_nat_* are the percentages of ^13^C measured in enriched and control samples respectively, *C_inc_* is the percentage of ^13^C enrichment of the seawater (which varies during the chase, see [39]), *M_sample_* is the mass of the sample (mg), *M_C_* is the carbon content per biomass of symbiont or host tissue (µg mg^−1^) or the mass of released POC (µg), S is the surface area of the nubbin (cm^2^), *t_pulse_* and *t_chase_* are the incubation times (h) in the enriched and non-enriched incubation media, respectively, in the light. See Table 2 for a list of symbols and their definitions.

The percentage of fixed carbon remaining (*C_R_*) in symbionts, host tissue, or POC is calculated by dividing *ρ_S_*, *ρ_H_*, or *ρ_POC_* by gross photosynthesis expressed in carbon (*P_C_*), and multiplying by 100.


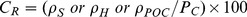
(2)

The carbon acquired through photosynthesis (*P_C_*) may have different fates, i.e. respiration by the coral assemblage (*R_C_*), incorporation into the biomass of the symbiont (*ρ_S_*) and the host (*ρ_H_*), and loss to the surrounding water as POC (*ρ_POC_*) and DOC (*ρ_DOC_*). Hence, the carbon budget equation is:
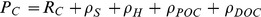
(3)


*R_C_* includes two components, i.e. respiration of the symbiont (*R_S_*) and the host (*R_H_*): *R_S_* + *R_H_* = *R_C_*.

Equation 3 does not consider the carbon incorporated in the skeleton because it has been shown that 25–30% comes directly from the external medium as dissolved inorganic carbon and 70–75%, from internal respiration *R_C_* [46], [47]; the first component is external to the equation, and the second component is part of *R_C_*. It follows from equation 3 that the amount of carbon lost as combined *R_C_* and *ρ_DOC_* (*C_L_*) is:


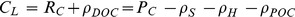
(4)

The amount of carbon translocated by symbionts to the host (*T_S_*) corresponds to the total amount of carbon gained by photosynthesis (*P_C_*) minus the sum of the carbon retained in symbionts (*ρ_S_*) and respired by them (*R_S_*):


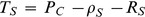
(5)

The percentages of carbon lost (*C_L_*) and translocated (*T_S_*) are obtained by dividing *C_L_* or *T_S_* by *P_C_*, and multiplying by 100.

In the present study, *ρ_DOC_* was not measured but estimated from the carbon lost equation (eq. 4):


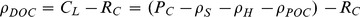
(6)

### Statistical Analysis

All parameters were expressed as average value ± standard error of the mean (s.e.m.). Data were checked for normality using a Kolmogorov-Smirnov’s test with Lilliefors correction, and for variance homoscedasticity using a Levene’s test. When the normality condition was not fulfilled, data were transformed (natural logarithms) or a nonparametric test was used. Preliminary partly nested analyses of variance (ANOVA) were used to test the tank effect, with three factors (irradiance, feeding mode, and tank). Because the tank effect nested within irradiance and feeding mode was not significant, it was not considered in further analyses. The two tanks per condition were pooled according to the procedure described in [48], to check that variation among experimental units was zero, and that pooling was appropriate and did not change the conclusion of the analysis. Significant differences for physiological parameters were tested using factorial ANOVA or nonparametric Sheirer-Ray-Hare’s test with two factors (irradiance, and feeding mode). The effect of treatments on the incorporation rates (*ρ*), *C_R_*, *C_L_*, and *T_S_*, after 0 and 48 h, was tested using a factorial ANOVA with two factors (irradiance, and feeding mode). When there were significant differences, the analyses were followed by *a posteriori* testing (Tukey’s test). Differences in the amount of carbon lost as combined respiration and DOC (*C_L_* = *R_C_ + ρ_DOC_*) and total respiration (*R_C_*) were tested using a *t*-test with seven degrees of freedom (*df* = *n_CL_* + *n_RC_* –2 = 3+6–2). Differences between factors were considered significant for *p*-values <0.05. Statistics were performed using Systat 13 (Systat Software, Chicago, IL, USA).

## Results

### Effects of Irradiance and Feeding on the Main Physiological Parameters

Several physiological parameters of colonies of *C. caespitosa* were significantly different according to the culture irradiance, independently of the feeding regime (Figure 1; Table 3). Chlorophyll concentration in symbiont cells and total protein content were significantly lower under HI (Figure 1C,D; Table 3). In contrast, other parameters were significantly higher under HI, i.e. calcification rate (Figure 1E; Table 3), C:N ratios of symbionts and host tissue (Figure 1F; Table 4), rate of gross photosynthesis, and symbiont respiration rate (Figure 2A,C; Table 5). Gross photosynthesis supplied three times more carbon to nubbins maintained under HI than LI (205 to 216 *versus* 56 to 78 µg C cm^−2^ d^−1^, respectively). Respiration consumed 120 to 166 *versus* 89 to 122 µg C cm^−2^ d^−1^ in nubbins under HI and LI, respectively.

Under both LI and HI, fed nubbins had significantly higher protein content (Figure 1D; Table 3), and lower C:N ratios of symbionts and host tissue (Figure 1F; Table 4) than unfed ones, indicating that fed corals were nitrogen-richer than unfed ones. Under HI, fed nubbins also had significantly higher symbiont concentration (Figure 1A; Table 3 and Tukey’s test *p* = 0.0005) and C:N ratio of released POC/PON (Figure 1F; Table 4) than unfed ones, the latter indicating higher excretion of carbon relative to nitrogen.

### Carbon Translocation between Symbionts and their Host

The natural atom %^13^C (measured in non-enriched control corals) was similar in all treatments (Table 4; atom %^13^C = 1.1326±0.0003 and 1.1297±0.0002%, for symbionts and coral host, respectively). After incubation in ^13^C-bicarbonate, all nubbins were richer in ^13^C compared to control corals (atom %^13^C between 1.2496 and 1.8865% in symbionts, and between 1.1440 and 1.3164% in the host tissue).

There were both independent and crossed effects of feeding and irradiance on the fate of the autotrophically-acquired carbon (Table 6). Incorporation rates in symbionts and host tissue at the beginning of the chase were two to four times higher under HI than LI (3 to 6 *versus* <1.5 µg C cm^−2^ h^−1^, respectively; Figure 3A,B; Table 6). Incorporation rates in symbionts remained quite constant during the whole chase under LI, whereas they decreased under HI (Figure 3A), suggesting carbon translocation to the host. This decrease was faster in symbionts of fed than unfed corals (Figure 3A). In the host fraction, incorporation rates remained constant under LI, but increased during the first 4 h under HI before decreasing until the end of the incubation (Figure 3B). Incorporation rate in the released POC was much higher for corals maintained under HI than LI (Figure 3C), although the amount was two orders of magnitude smaller than carbon incorporation in symbionts (Figure 3A) and the host tissue (Figure 3B).

At the end of incubation (48 h), depending on the treatment, only 10 to 22% (i.e. 0.7 to 3.4 µg C cm^−2^ h^−1^) of the total fixed carbon remained in symbionts or the host tissue, (Figure 3A,B and 4A,B). Total losses accounted for 44 to 67% of the carbon fixed at the beginning of the chase, and reached 62 to 76% at the end of the chase (Figure 5B). Over the 48-h chase, between 3 and 5 µg C cm^−2^ h^−1^ were lost by nubbins maintained under LI, and between 11 and 14 µg C cm^−2^ h^−1^ by nubbins under HI (Figure 5A). Less than 0.4% of the total fixed carbon was released as POC (Figure 4C), indicating that losses were essentially in the form of respiration and DOC. Carbon loss (Figure 5A) was similar to respiration rates (Figure 2B) in nubbins maintained under LI (LIF *t*-test *p* = 0.3839; *t* = 0.93; *df* = 7 and LIU *t*-test *p* = 0.8178; *t* = 0.24; *df* = 7), but was much higher in nubbins under HI (HIF *t*-test *p* = 0.0034; *t* = 4.33; *df* = 7 and HIU *t*-test *p* = 0.0004; *t* = 6.30; *df* = 7). These results indicate that respiration was responsible for most of the carbon loss under LI, whereas release of newly-fixed carbon as DOC accounted for a significant fraction of carbon loss under HI. The latter was ca. 5.9 and 6.8 µg C cm^−2^ h^−1^ or 34–37% of the fixed carbon in unfed and fed nubbins, respectively.

The amount of carbon translocated after 48 h was significantly higher under HI (ca. 13 µg C cm^−2^ h^−1^) than under LI (Figure 6A; Table 6 and Tukey’s test *p*<0.0001) because rates of photosynthesis and the total carbon fixed under HI were also higher (Figure 2A). However, the percentage of total fixed carbon translocated was independent of the irradiance under which nubbins were maintained, and reached a maximum value of 70–74% (Figure 6B). Overall, feeding had a negative effect on translocation rates. Indeed, fed nubbins maintained under LI showed a significantly lower amount of carbon translocation per surface area than unfed nubbins (2.5 *versus* 4.5 µg C cm^−2^ h^−1^; Figure 6A; Table 6 and Tukey’s test *p* = 0.0021) and lower percentage of carbon translocated (53 *versus* 70%; Figure 6B; Table 6 and Tukey’s test *p* = 0.0155). Under HI, translocation was also slower in fed than in unfed nubbins, with 70% translocation being reached after 24 h in fed nubbins against a few hours only in unfed nubbins (Figure 5D). Finally, carbon translocation rate per symbiont cell was two times lower in fed nubbins (3.9±0.1 and 1.4±0.1×10^−6^ µg C cell^−1^ h^−1^ for HI and LI, respectively) than unfed nubbins (6.5±0.2 and 2.7±0.1×10^−6^ µg C cell^−1^ h^−1^ for HI and LI, respectively; not shown in a Figure).

We used the results from our experiments under the four combinations of irradiance and feeding conditions to build two contrasted autotrophic carbon budgets (Figure 7). The differences observed between low and high irradiances will be discussed below.

## Discussion

This study assessed the fate of the photosynthetically-acquired carbon within a temperate coral symbiosis by tracing the pathways of ^13^C-enriched photosynthates in the symbionts, the coral host tissue, and the particulate organic compounds released by the association. Our approach identified novel aspects in the functioning of this temperate symbiosis, which are summarised here and developed in the following paragraphs. Firstly, symbionts had high percent translocation even when maintained under low irradiance, which was contrary to our first hypothesis (see the Introduction). However, the amount of carbon translocated was higher under high than low irradiance. Secondly, the host nutritional status affected carbon translocation, which was lower when the host was fed. This was in agreement with our second hypothesis. Thirdly, the fate of the photosynthesized carbon was different according to irradiance: it was respired by the symbiotic association under low irradiance, and both respired and lost as DOC under high irradiance, the latter suggesting that temperate corals produce carbon in excess of their metabolic requirements under high light. Overall, our results elucidate the roles of symbionts and heterotrophic feeding in the supply of carbon to the temperate coral host at two contrasted irradiances.

Our first hypothesis was that carbon translocation rates would be low under low irradiance because of carbon limitation of the symbionts. Only two studies, to our knowledge, have assessed carbon translocation rates in temperate symbioses at different irradiances, and these had been conducted on sea anemones, not corals. In *Anthopleura elegantissima* maintained at 10 and 100 µmol photons m^−2^ s^−1^, Engebreston and Muller-Parker [38] observed no effect of irradiance on percentages of translocation, which were approximately 50% under both irradiances. In contrast, Davy et al. [36] observed, for three species of sea anemones, lower percent translocation under low than high irradiance, i.e. values ranging between 28 and 75% at 10 µmol photons m^−2^ s^−1^ compared to >86% at higher irradiance. In the present study and considering only unfed nubbins on which the effect of irradiance can be tested independently from that of feeding, carbon translocation reached ca. 70% of the total carbon fixed by photosynthesis after 48 h, at both 20 and 120 µmol photons m^−2^ s^−1^. The amount of carbon translocated, however, was two to three times lower under low than high irradiance because of light limitation of photosynthesis. Our results indicate that, under very low irradiance, symbionts of unfed *C. caespitosa* did not keep all the fixed carbon for their own use despite the low amount produced. Indeed, they only kept the amount needed for their respiration (16% of 6.5 µg C cm^−2^ h^−1^ photosynthesized carbon correspond to 1 µg C cm^−2^ h^−1^ respired, Figure 7A), plus a small amount incorporated in their biomass (1 µg C cm^−2^ h^−1^). This is contrary to our first hypothesis of low percent carbon translocation under low irradiance. The high percent carbon translocation under low irradiance, together with the maintenance of a high symbiont concentration, seems to be a strategy to optimize autotrophic carbon acquisition under low irradiance, as proposed by previous studies [15], [33].

There was however a different allocation of the photosynthetic carbon acquired by unfed nubbins according to the culture irradiance. Under low irradiance, a large fraction of this carbon was respired shortly after its production (Figure 7A; production of POC was negligible, and that of DOC was no significant), as already observed for sea anemones and tropical corals [10], [49]–[51]. This result shows the preferential use of photosynthates for respiration as opposed to longer-term storage [11], and suggests that under low irradiance, autotrophic carbon was used to sustain the basic metabolism of *C. caespitosa*. Under high irradiance on the contrary, 17% of the carbon photosynthesized by unfed nubbins was incorporated into symbiont biomass (17% of 17 µg C cm^−2^ h^−1^, or ca. 3 µg C cm^−2^ h^−1^, Figure 7B), although the respiration rates were twice as high as under low irradiance (ca. 2 µg C cm^−2^ h^−1^). This carbon was likely used as carbon-rich symbiont reserves, such as lipids, as indicated by the significantly higher C:N ratio of symbionts under high than low irradiance. The remainder of the acquired carbon was translocated to the host, where 18% was respired, 20% incorporated into the host biomass, and 34% released as DOC. The percentage of photosynthetic carbon released as POC was very low under the two incubation irradiances. The present study is the first to estimate the loss of fixed carbon as DOC and/or POC in temperate corals, but the percentage of DOC release we determined under high irradiance was within the range of 6–70% previously determined for tropical species [9], [11]–[13], [52]–[54]. Our results indicate that temperate corals can produce carbon in excess of their metabolic and growth requirements, at least under high irradiance, and release the excess organic carbon to the surrounding medium.

Concerning the fate of the respired carbon, it is usually assumed, at least for tropical corals, that 70 to 75% of the carbon used in calcification comes from respiration [46], [47]. In the present study, the rates of respiration could entirely fulfil calcification in all conditions. Under high irradiance, the measured calcification rates of 2.4 to 2.9 µg C cm^−2^ h^−1^ indeed corresponded to 74 to 77% of the host respiration (which was 3.1 to 3.9 µg C cm^−2^ h^−1^; Figure 7B). Under low irradiance, the measured calcification rates corresponded to only 22 to 42% of the host respiration (Figure 7A), suggesting that under this condition, calcification was limited by some other factors. The different factors that enhance calcification under high irradiance (or repress it in darkness) have been reviewed in several papers [55], [56].

The second hypothesis tested in this study was low carbon translocation rates when the host was fed, because it could then acquire enough heterotrophic carbon to be at least partly independent of the photosynthetic carbon. The effect of feeding on carbon translocation rates has been tested only once, to our knowledge, on the sub-tropical sea anemone *Aiptasia pallida* [57]. In this latter study, no effect of feeding was found on percent translocation, which was, however, very low (16%). In the present study, the amount of carbon translocated per symbiont cell was lower in fed than unfed nubbins under both irradiances (40 to 50% decrease, see the Results section), indicating that the carbon demand of the host was lower per symbiont cell. Lower amount of carbon translocated per surface area, as well as a lower percent translocation, were also observed under low irradiance. Under high irradiance, although translocation rate per symbiont cell was lower in fed than unfed corals, translocation rate per surface area was the same for the two feeding conditions because fed corals had higher symbiont concentration (Figure 1A), as also observed by Davy and Cook [57]. Overall, these results are in agreement with our second hypothesis, suggesting that the acquisition of autotrophic carbon by the host is reduced when it has access to heterotrophic carbon.

Results also show a different effect of heterotrophic feeding depending on the incubation irradiance. Under low irradiance, heterotrophy was accompanied by higher host biomass than in unfed corals (significantly higher protein content, Figure 1D), and there was no effect on symbiont concentration (Figure 1A). The same occurred under high irradiance (higher protein content in fed than unfed corals, Figure 1D), but symbiont concentration was also higher in fed than unfed corals (Figure 1A). Hence, heterotrophic feeding influenced the holobiont and not only the host.

The carbon budgets in Figure 7 contrast the allocation of autotrophic carbon under the two culture irradiances. These budgets cannot be applied directly to seasonal changes in the natural environment because the latter presents an additional level of complexity, i.e. co-variation of water temperature with irradiance, the two factors influencing holobiont physiology. Temperature used in the present experiments (18.0°C) was higher than *in situ* temperatures during winter. The purpose of this study was not to obtain budgets that would reproduce what happens seasonally in nature, but to disentangle the relative effects of irradiance and feeding on autotrophy in temperate symbioses. Our results showed that symbionts are of greater benefit to the host under “summer” than “winter” conditions, but can still provide significant carbon inputs to the association even under very low irradiance.

In summary, our results show that under low irradiance, high symbiont concentration and high percentage of carbon translocation allowed corals to optimize their acquisition of autotrophic carbon under unfavourable conditions. Most of this carbon was respired to satisfy the basic metabolic requirements of the holobiont, and almost none was lost to the surrounding medium. Under such conditions, heterotrophy was used to build the host biomass. Under high irradiance, which only occurs in summer in temperate areas, autotrophic carbon production was higher than under low irradiance, and mostly used to sustain respiration and high calcification rates. The production of carbon exceeded its use by more than 30%, the excess being lost to the surrounding medium as DOC. Heterotrophic feeding influenced the biomasses of the coral host and the symbionts. Our results largely elucidate the functioning of a temperate coral symbiosis, and the role of symbionts in the carbon budget of their coral host.
